# The population-level effects of omitting chemotherapy guided by a 21-gene expression assay in node-positive breast cancer: a simulation modeling study

**DOI:** 10.1186/s12885-024-12719-3

**Published:** 2024-08-08

**Authors:** Kaitlyn M. Wojcik, Jennifer L. Caswell-Jin, Oliver W.A. Wilson, Clyde Schechter, Dalya Kamil, Allison W. Kurian, Jinani Jayasekera

**Affiliations:** 1 Health Equity and Decision Sciences Laboratory, National Institute on Minority Health and Health Disparities, Division of Intramural Research, National Institutes of Health, Bethesda, MD USA; 2 Department of Medicine, Stanford University School of Medicine, Stanford, CA USA; 3 Department of Family & Social Medicine at Albert Einstein College of Medicine, Bronx, NY USA; 4 Department of Epidemiology and Population Health, Stanford University School of Medicine, Stanford, CA USA

## Abstract

**Background:**

A recent trial showed that postmenopausal women diagnosed with hormone receptor-positive, human epidermal growth factor receptor-2 (HER2)-negative, lymph node-positive (1–3 nodes) breast cancer with a 21-gene recurrence score of ≤ 25 could safely omit chemotherapy. However, there are limited data on population-level long-term outcomes associated with omitting chemotherapy among diverse women seen in real-world practice.

**Methods:**

We adapted an established, validated simulation model to generate the joint distributions of population-level characteristics of women diagnosed with early-stage breast cancer in the U.S. Input parameters were derived from cancer registry, meta-analyses, and clinical trial data. The effects of omitting chemotherapy on 10-year distant recurrence-free survival, life-years, and quality adjusted life-years (QALYs) were modeled for premenopausal and postmenopausal women. QALYs were discounted at 3%. Results were evaluated for subgroups stratified by race and ethnicity. Sensitivity analyses included testing results across a range of inputs. The model was validated using the published RxPONDER trial data.

**Results:**

In premenopausal women, the 10-year distant recurrence-free survival rates were 85.3% with chemo-endocrine and 80.1% with endocrine therapy. The estimated life-years and QALYs gained with chemotherapy in premenopausal women were 2.1 and 0.6, respectively. There was no chemotherapy benefit in postmenopausal women. There was no variation in the absolute benefit of chemotherapy across racial or ethnic subgroups. However, there were differences in distant recurrence-free survival rates, life-years, and QALYs across groups. Sensitivity analysis showed similar results. The model closely replicated the RxPONDER trial.

**Conclusions:**

Modeled population-level outcomes show a small chemotherapy benefit in premenopausal women, but no benefit among postmenopausal women. Simulation modeling provides a useful tool to extend trial data and evaluate population-level outcomes.

**Supplementary Information:**

The online version contains supplementary material available at 10.1186/s12885-024-12719-3.

## Introduction

The recent ‘Rx for Positive Node, Endocrine Responsive Breast Cancer’ (RxPONDER) trial found that postmenopausal women diagnosed with hormone receptor positive (HR+), human epidermal growth factor 2-negative (HER2-), node-positive breast cancer with a 21-gene recurrence score (RS) of ≤ 25 could safely omit chemotherapy, while a chemotherapy benefit was observed among premenopausal women at all RS levels [1]. These results may mean that many women with HR+/HER2- breast cancer, the most common subtype, could safely avoid chemotherapy, reducing their financial burden as well as exposure to treatments that may have significant side effects [2–5].

However, there were several questions left unanswered by the RxPONDER trial [1]. First, the trial did not provide information on breast cancer outcomes beyond five-year disease- and distant relapse-free survival, such as life-years gained, quality adjusted life-years (QALYs), and breast cancer death [6]. Information about QALYs, adjusted to an individual patient’s preferences, could provide broad information about the quality of life, in addition to the quantity, when comparing available treatment options [7, 8]. Moreover, clinical trial data may have limited generalizability to real-world populations and conditions [9]. RxPONDER was a single trial evaluating the impact of chemotherapy in ideal conditions; as a result, there is limited data on the possible variation of trial results in real-world settings [10]. Finally, the majority of women who participated in the RxPONDER trial were of White race (85% of those whose race was not classified as “Other/Unknown”) and non-Hispanic ethnicity (85% of those whose ethnicity was not classified as “Other/Unknown”) [1]. As a result, the trial had limited information on the possible variation of chemotherapy effects in non-Hispanic Black and Hispanic women. A recent post-hoc analysis of the RxPONDER trial reported that Black women had lower 5-year distant relapse–free survival rates compared to non-Hispanic White women [11]. However, the post-hoc analyses provided limited information on long-term, population-level outcomes associated with chemotherapy in non-Hispanic Black, Hispanic, and non-Hispanic White women.

In the context of these limitations, simulation modeling can be used to extend trial results further than what was available at the time of the original trial [12]. Additionally, consistent results across clinical trials and simulation models could increase physicians’ confidence when making treatment decisions with their patients [13]. Simulation modeling could also be used to estimate the population-level impact of trial findings, variation of results across population subgroups, and outcomes not evaluated in the original trial, such as life-years and QALYs [7, 14].

In this study, we adapted an existing, validated, discrete-event simulation model to extend the published RxPONDER trial data to estimate long-term, population-level breast cancer outcomes in women diagnosed with HR+, HER2-, node-positive (1–3 nodes) breast cancer with a RS of ≤ 25 [14, 15]. The overarching goal of this study was to extend trial results to estimate population-level outcomes and support the integration of trial findings into clinical practice.

## Methods

This study was approved by the National Institutes of Health Institutional Review Board and was considered as exempt research based on use of de-identified pre-existing data (IRB001806).

### Overview of simulation model

We adapted a previously developed and validated discrete-event model of breast cancer [14, 15]. This model has been used to simulate clinical trial protocols, including ‘The Trial Assigning Individualized Options for Treatment’ (TAILORx) [14, 16]. Model details are provided in the Data Supplement. For this study, the model was adapted to replicate the RxPONDER trial protocol and was extended to estimate the population-level effects of omitting chemotherapy in women diagnosed with HR+, HER2-, node-positive (N1, 1–3 nodes) breast cancer with a RS of ≤ 25. Distant recurrence rates, life-years gained, and quality adjusted life-years (QALYs) for endocrine and chemo-endocrine therapy were generated for the overall population and women stratified by menopausal status.

### Modeled population

The model simulated female patients aged ≥ 18 years, diagnosed with HR+, HER2-, node-positive (1–3 nodes) breast cancer (without distant metastasis) with a RS of ≤ 25 who had undergone surgery and were eligible to receive chemo-endocrine therapy or endocrine therapy only [1]. We generated virtual samples of simulated patients with unique combinations of age, tumor grade, tumor size, estrogen (ER) and progesterone receptor (PR) status, menopausal status, and RS from the joint frequency distribution of these characteristics as a simple random sample in the overall population stratified by race and ethnicity.

### Modeled breast cancer outcomes

Simulated patients were randomly assigned in a 1:1 ratio to receive endocrine or chemo-endocrine therapy and then followed until death. Simulated patients could experience one of four possible outcomes depending on their combination of unique characteristics and treatment: remain event free, experience distant recurrence, die of breast cancer, or die of other causes. The primary endpoint of interest was distant recurrence-free survival (DRFS) at 10-years, defined as time from diagnosis to date of distant recurrence, or death with distant recurrence, if death was the first manifestation of distant recurrence. This definition corresponds to the Standardized Definitions for Efficacy End Points (STEEP) in Adjuvant Breast Cancer Trials definition of distant recurrence-free survival [17].

### Model inputs

All input parameters and data sources are listed in Table 1. The overall and race and ethnicity-specific population-level joint distributions of age at diagnosis, tumor grade, tumor size, ER/PR status, and RS were derived from the Surveillance, Epidemiology, and End Results (SEER) Program database [18]. Since SEER does not record menopausal status, the joint distribution of age and menopausal status overall and by race and ethnicity were simulated using published TAILORx trial data [19]. The derivation of input parameters for age and tumor characteristics are described in detail in Supplemental Methods and Supplemental Table 1. Time-to-events (distant recurrence, breast cancer death, and other cause death) were generated conditional on age, menopausal status, tumor size, grade, treatment, RS, race, and ethnicity [14, 19–21]. Previously generated input parameters for time-to-events using NSABP B-14/B-20 and TAILORx trials [14] were adjusted using published data from the TransATAC trial [22] for endocrine therapy in women diagnosed with hormone receptor positive, node positive (nodes 1–3) breast cancer and recurrence scores ranging from 0 to 25. The chemotherapy effects were adjusted according to a meta-analysis of clinical trial data for early-stage breast cancer [23]. Therefore, the time-to-event input parameters for the current model were generated from data independent of the RxPONDER trial. We assumed 100% treatment adherence to isolate the variation of chemotherapy effects in subgroups stratified by menopausal status. Utilities estimated for the average person associated with age, diagnoses, treatment, side-effects, and distant recurrence were derived from published data [21, 24–32].

**Table 1:** Input parameters derived for women diagnosed with node-positive (1–3 nodes), hormone receptor positive, HER2 negative breast cancer with a 21-gene recurrence score of ≤ 25

Characteristics	Description	Data Source
Age distribution	Distribution of age in women diagnosed with node-positive (1–3 nodes), hormone receptor positive, HER2 negative breast cancer by race and ethnicity.	SEER [18]
Menopausal status	Conditional on woman’s age, race, and ethnicity.	Published trial data [19]
Tumor grade	Conditional on a woman’s age, race, and ethnicity.	SEER [18]
ER/PR status	Conditional on the woman’s tumor grade, race, and ethnicity.	SEER [18]
Tumor size	Conditional on tumor grade, woman’s age, race, and ethnicity.	SEER [18]
21-gene-recurrence score	21-gene-recurrence score conditional on age, tumor grade, tumor size, ER/PR status, race, and ethnicity.	Genomic Health (published data) [33]
Other cause mortality	Other cause mortality rates by age, race, and ethnicity.	CDC WONDER [34]
Events and Event times	Conditional on age, tumor grade, tumor size, treatment, 21-gene recurrence score, menopausal status, race, ethnicity, and treatment.	Published clinical trial data [14, 19, 20, 22, 23]
	**Probabilities**	
Grade 3, 4 Toxicity	0.175 (0.17–0.20)	Published data [21, 24–28]
	**Utilities**	
Age		Published data [29, 30]
20–29	0.913 (0.905–0.920)	
30–39	0.893 (0.886–0.900)	
40–49	0.863 (0.855–0.871)	
50–59	0.837 (0.829–0.846)	
60–69	0.811 (0.800–0.822)	
70–79	0.771 (0.758–0.784)	
Invasive breast cancer with surgery (mastectomy or breast-conserving surgery), radiation therapy, and/or chemotherapy (AJCC 7th Stage I, II)	0.731 (SD 0.255)	Published data [31, 32]
Chemotherapy	0.9 (6-mo duration)	Published data [31, 32]
Grade 3–4 toxicity	0.7 (6-mo duration)	Published data [31, 32]
Distant recurrence	0.4 (3 years)	Published data [31, 32]

*Includes Black non-Hispanic, Hispanic, and non-Hispanic White

AJCC: American Joint Committee on Cancer; CDC WONDER: Centers for Disease Control and Prevention Wide-ranging Online Data for Epidemiologic Research

ER Estrogen Receptor; PR Progesterone Receptor; RxPONDER: Rx for Positive Node, Endocrine Responsive Breast Cancer; SEER: Surveillance, Epidemiology, and End Results Program

### Statistical analysis

We used an empirical Bayesian analytical approach to capture the uncertainty in all predictors’ effects (i.e., treatment, age, tumor grade, tumor size, recurrence score, race, ethnicity, menopausal status) on outcomes and sampling variation. The overall population and subgroups were randomly assigned their own set of treatment effects sampling from the “prior” distribution of the sub-hazard ratios derived from the competing risk survival models. The 5- and 10-year distant recurrence rates and breast cancer death rates were estimated using Kaplan-Meier curves. The incremental differences comparing chemo-endocrine vs. endocrine therapy were calculated for the overall population and women stratified by menopausal status.

We assigned utility values based on the national female population age-specific values for general health from the EQ-5D reported in the Medical Expenditure Panel Survey data [21, 24–28]. Utilities were further adjusted for early-stage breast cancer, chemotherapy use and toxicity, and distant recurrence [29–32]. Since all patients were assumed to receive endocrine therapy, the disutility for endocrine therapy was not included. The sum of life-years and incremental life-years comparing chemo-endocrine vs. endocrine therapy were calculated for women stratified by menopausal status. QALYs were obtained from the sum of life-year(s) multiplied by the utility value for each event. QALYs were discounted at 3% for each treatment strategy (chemo-endocrine and endocrine therapy). A positive incremental QALY indicated that chemotherapy benefits outweighed its harms, while the negative values indicated that toxicity harms were greater than benefits [7]. The incremental differences comparing chemo-endocrine vs. endocrine therapy were calculated for the overall population and women stratified by menopausal status.

### Subgroup analysis

Results for distant recurrence-free survival rates, life years, and QALYs were generated for non-Hispanic Black, non-Hispanic White, and Hispanic premenopausal and postmenopausal women.

### Sensitivity analysis

Sensitivity analyses were conducted to examine the effect of varying the input parameters on model outcomes. First, the results were generated using age 50 as a proxy for menopausal status. Second, we varied the probabilities of chemotherapy toxicity across the 95% confidence interval (CI). Finally, to evaluate the effects of time preferences on outcomes, QALYs were also discounted at alternative rates of 1% and 5% per year.

#### Model validation

The model was validated by simulating the RxPONDER trial using data independent of the trial. The specifications for the detection of relative differences in the effects of endocrine vs. chemo-endocrine therapy on distant recurrence were used to set the sample size for the virtual trial. We assumed a null hypothesis of no difference between the two treatment arms [1]. The comparison of patient and tumor characteristics in the simulated and actual RxPONDER trials are provided in Supplemental Tables 2–3.

All analyses were conducted using Stata, version 18.0 (StataCorp. 2023. Stata Statistical Software: Release 18. College Station, TX: StataCorp LLC) [35].

## Results

### Simulated participant characteristics

We simulated a total of five-million women. The median age of the overall sample was 57.4 years, and 28.6% of the women were premenopausal. The majority of the tumors were intermediate grade (57.8%) and positive for both hormone receptors (ER and PR) (85.4%) (Table 2).

**Table 2:** Patient and tumor characteristics of the simulated women diagnosed with node-positive (1–3 nodes), hormone receptor positive, HER2 negative breast cancer with 21-gene recurrence scores of ≤ 25 stratified by menopausal status and receipt of endocrine vs. chemoendocrine therapy

Characteristics	Overall		Premenopausal		Postmenopausal	
	Endocrine therapy (Col %)	Chemo-endocrine therapy (Col %)	Endocrine therapy (Col %)	Chemo-endocrine therapy (Col %)	Endocrine therapy (Col %)	Chemo-endocrine therapy (Col %)
Age at Diagnosis (median, years)	57.4	57.4	45.2	45.3	62.4	62.4
**Menopausal status**						
Premenopausal	28.6	28.7	100.0	100.0	0.0	0.0
Postmenopausal	71.4	71.3	0.0	0.0	100.0	100.0
**Recurrence score**						
Median	17.0	17.0	18.0	18.0	17.0	17.0
**Tumor Grade**						
Low	27.0	27.2	24.3	24.4	28.4	28.2
Intermediate	57.8	57.7	59.0	59.0	57.3	57.3
High	15.2	15.1	16.7	16.6	14.4	14.5
**Hormone Sensitivity**						
ER and PR positive	85.4	85.4	83.7	83.7	86.2	86.1
ER or PR positive	14.6	14.6	16.3	16.3	13.8	13.9
**Tumor size**						
2 cm or less	53.2	53.2	52.7	52.9	53.5	53.5
Greater than 2 cm	46.8	46.8	47.3	47.1	46.5	46.5

ER: estrogen receptor; PR: progesterone receptor; HER2: Human epidermal growth factor receptor 2

### Distant recurrence-free survival rates

**Fig. 1:**
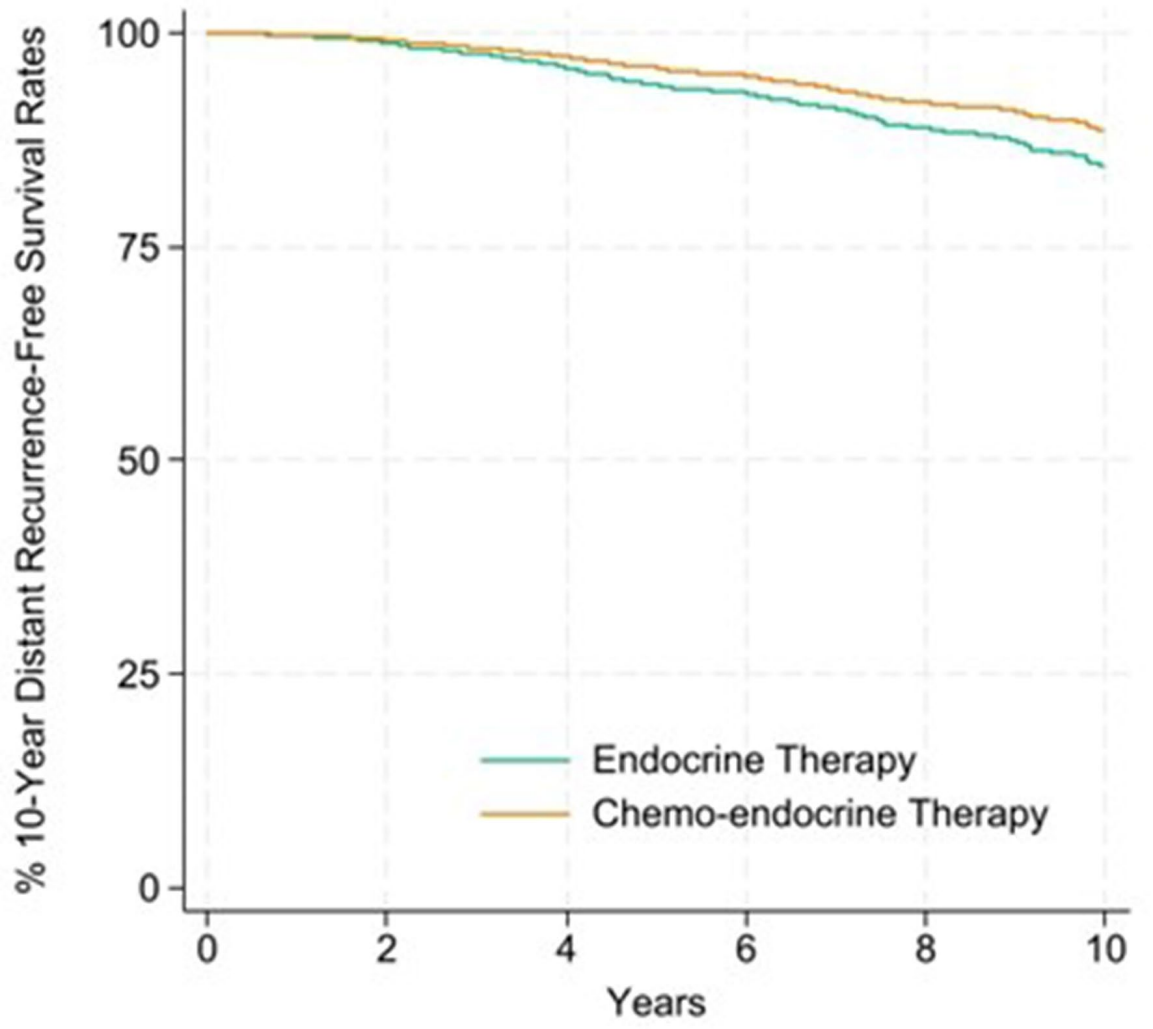
Kaplan-Meier curve for the percentage of 10-year distant recurrence-free survival rates for premenopausal women

**Fig. 2:**
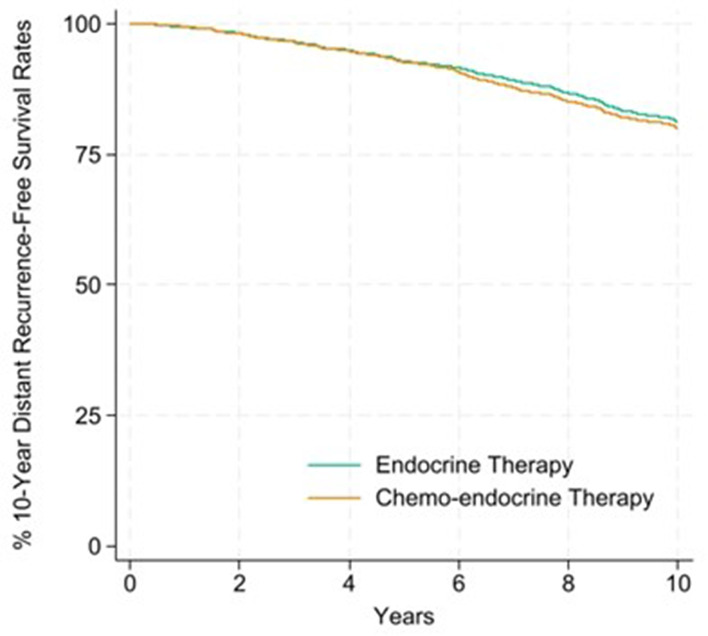
Kaplan-Meier curve for the percentage of 10-year distant recurrence-free survival rates for postmenopausal women

Overall, the 10-year distant recurrence-free survival rate was 81.9% for chemo-endocrine therapy and 81.5% for endocrine therapy, with an absolute chemotherapy benefit of 0.4% points (%pts) (Table 3). In premenopausal women, the 10-year distant recurrence-free survival rate was 85.3% for chemo-endocrine therapy and 80.1% for endocrine therapy, with an absolute benefit of 5.6% points. There was no chemotherapy benefit in postmenopausal women. Breast cancer-free survival rates are provided in Supplemental Table 3. Kaplan-Meier curves detailing distant recurrence-free survival up to 10 years for premenopausal and postmenopausal women are shown in Figs. 1 and 2.

**Table 3:** Simulation model results for distant recurrence-free survival at 5- and 10-years for endocrine vs. chemoendocrine therapy for women diagnosed with node positive (1–3 nodes), hormone receptor positive, HER2 negative breast cancer with 21-gene-recurrence scores of ≤ 25, overall and stratified by menopausal status

	5-year Distant Recurrence-Free Survival Rates (%)			10-year Distant Recurrence-Free Survival Rates (%)		
	Endocrine therapy	Chemo-endocrine therapy	Absolute Difference^1^	Endocrine therapy	Chemo-endocrine therapy	Absolute Difference^1^
**Overall**	93.1	93.2	0.1	81.5	81.9	0.4
**Premenopausal**	92.4	94.5	2.2	79.7	85.3	5.6
**Postmenopausal**	93.5	92.7	-	82.4	80.1	-

^1^Chemo-endocrine therapy (minus) Endocrine therapy Rates

### Life-years and QALYs

Overall, approximately 0.6-life years and 0.4–0.5 QALY losses were estimated due to chemotherapy. In premenopausal women, the life-years gained with chemotherapy were approximately 2.1 years. There was a small, estimated increase in QALYs (0.6–1.1) with chemotherapy for premenopausal women. In postmenopausal women, there were life-years (2.0), and QALY (1.0-1.3) losses due to chemotherapy (Table 4).

**Table 4:** Simulation model results for life-years and 3% discounted quality-adjusted life-years (QALYs) for endocrine vs. chemoendocrine therapy for women diagnosed with node positive (1–3 nodes), hormone receptor positive, HER2 negative breast cancer with 21-gene-recurrence scores of ≤ 25, overall and stratified menopausal status

	Life Years			Undiscounted QALYs			3% Discounted QALYs		
	Endocrine therapy	Chemo-endocrine therapy	Life Years Gained^1^	Endocrine therapy	Chemo-endocrine therapy	QALYs Gained^1^	Endocrine therapy	Chemo-endocrine therapy	QALYs Gained^1^
**Overall**	19.6	19.0	-0.6	12.4	11.9	-0.5	10.1	9.7	-0.4
**Premenopausal**	27.8	29.9	2.1	17.6	18.8	1.1	13.6	14.2	0.6
**Postmenopausal**	15.4	13.4	-2.0	9.7	8.4	-1.3	8.4	7.4	-1.0

^1^Chemo-endocrine therapy (minus) Endocrine therapy Life-years or QALYs

### Subgroup analysis

The SEER-based clinical and demographic characteristics of women, stratified by race and ethnicity, are provided in Supplemental Table 2. Overall, the average ages at diagnosis were 56.3 for non-Hispanic Black, 53.6 for Hispanic, and 58.0 for non-Hispanic White women. Consistent with SEER data [18], there were higher percentages of non-Hispanic Black women with high grade tumors (26.2–26.4%) and lower hormonal sensitivity (i.e., ER or PR+, rather than both ER and PR+) (21.7–21.8%) (Supplemental Table 2) compared to the overall population (high grade tumors (15.1–15.2%), and lower hormonal sensitivity (14.6%)) (Table 2).

The absolute benefits of chemotherapy stratified by race and ethnicity were similar to the results in the overall population, where a chemotherapy benefit was seen among premenopausal women, while no benefit was seen among postmenopausal women (Tables 5 and 6). However, distant recurrence-free survival rates, life-years, and QALYs in each treatment arm varied by race and ethnicity. For example, in the overall sample, the 10-year distant recurrence-free survival rates and life-years with chemo-endocrine therapy were 74.8% and 11.5-years in non-Hispanic Black, 76.7% and 17.3-years in Hispanic, and 83.2% and 19.9-years in non-Hispanic White women (Table 5).

**Table 5:** Subgroup analysis results for distant recurrence-free survival at 5- and 10-years for endocrine vs. chemoendocrine therapy for women diagnosed with node positive (1–3 nodes), hormone receptor positive, HER2 negative breast cancer with 21-gene-recurrence scores of ≤ 25, overall and stratified by race, ethnicity, and menopausal status

Race and Ethnicity	5-year distant recurrence-free survival rates			10-year distant recurrence-free survival rates		
	Endocrine therapy	Chemo-endocrine therapy	Absolute Difference^1^	Endocrine therapy	Chemo-endocrine therapy	Absolute Difference^1^
**All**						
Black (non-Hispanic)	89.8	90.0	0.2	74.6	74.8	0.3
Hispanic	90.3	90.4	0.1	76.7	76.7	0.0
White (non-Hispanic)	93.8	93.9	0.1	82.7	83.2	0.5
**Premenopausal**						
Black (non-Hispanic)	88.4	91.3	2.9	73.0	79.0	6.0
Hispanic	91.9	94.1	2.2	80.9	85.3	4.4
White (non-Hispanic)	92.8	94.9	2.2	80.2	85.9	5.7
**Postmenopausal**						
Black (non-Hispanic)	90.5	89.3	-	75.3	72.7	-
Hispanic	89.5	88.4	-	74.8	72.4	-
White (non-Hispanic)	94.3	93.5	-	84.0	81.8	-

^1^Chemo-endocrine therapy (minus) Endocrine therapy Rates

**Table 6:** Subgroup analysis results for life-years and 3% discounted quality-adjusted life-years (QALYs) for endocrine vs. chemoendocrine therapy for women diagnosed with node positive (1–3 nodes), hormone receptor positive, HER2 negative breast cancer with 21-gene-recurrence scores of ≤ 25, overall and stratified by race, ethnicity, and menopausal status

Race and Ethnicity	Life-Years			Undiscounted QALYs			3% Discounted QALYs		
	Endocrine Therapy	Chemo-endocrine Therapy	Life-Years Gained^1^	Endocrine Therapy	Chemo-endocrine Therapy	QALYs Gained^1^	Endocrine Therapy	Chemo-endocrine Therapy	QALYs Gained^1^
**All**									
Black (non-Hispanic)	11.7	11.5	-0.2	7.4	7.3	-0.2	6.6	6.5	-0.2
Hispanic	17.9	17.3	-0.6	11.4	10.9	-0.5	9.4	9.0	-0.4
White (non-Hispanic)	20.6	19.9	-0.6	13.0	12.5	-0.5	10.6	10.1	-0.5
**Premenopausal**									
Black (non-Hispanic)	15.1	16.7	1.6	9.6	10.6	1.0	8.3	9.0	0.7
Hispanic	27.7	29.2	1.6	17.6	18.4	0.8	13.6	14.1	0.5
White (non-Hispanic)	29.1	31.3	2.2	18.4	19.6	1.2	14.1	14.7	0.6
**Postmenopausal**									
Black (non-Hispanic)	10.0	8.9	-1.1	6.3	5.6	-0.8	5.8	5.1	-0.7
Hispanic	12.9	11.2	-1.7	8.3	7.1	-1.2	7.3	6.4	-0.9
White (non-Hispanic)	16.2	14.1	-2.1	10.2	8.8	-1.4	8.8	7.7	-1.1

^1^Chemo-endocrine therapy (minus) Endocrine therapy Life-years or QALYs

### Sensitivity analysis

The results were similar when using age 50 as a proxy for menopausal status (Supplemental Tables 4 and 5), varying rates of chemotherapy toxicity (Supplemental Table 6), and using alternate discount rates of 1 and 5% for QALYs (Supplemental Table 7).

### Model validation

Simulated patients in each treatment arm had a similar distribution of characteristics (age, tumor grade, tumor size, ER/PR status, and RS) to the women enrolled in the actual RxPONDER trial (Supplemental Tables 8 and 9). The model closely replicated RxPONDER trial results for the overall sample and women stratified by menopausal status (Table 7).

**Table 7:** Model validation

	5-Year distant recurrence-free survival rates (%)								
	Overall			Premenopausal			Postmenopausal		
	Endocrine therapy	Chemo-endocrine therapy	Absolute Difference^1^	Endocrine therapy	Chemo-endocrine therapy	Absolute Difference^1^	Endocrine therapy	Chemo-endocrine therapy	Absolute Difference^1^
RxPONDER Trial	93.9	94.9	1.0	92.8	96.1	3.3	94.4	94.4	0.0
Trial Simulation	93.1	94.5	1.3	92.4	96.0	3.6	94.2	94.5	-

RxPONDER: Rx for Positive Node, Endocrine Responsive Breast Cancer

^1^Chemo-endocrine therapy (minus) Endocrine therapy Rates

## Discussion

This study adds to the growing body of literature that uses simulation models to address health outcomes unexplored in clinical trials. Simulation modeling has been used to expand clinical trial outcomes for radiation and chemotherapy in node negative, HR+/HER2- breast cancer patients [14, 15]. However, to our knowledge, this is the first study that has used simulation modeling to expand clinical trial outcomes to analyze breast cancer outcomes stratified by menopausal status, race, and ethnicity in node-positive early-stage breast cancer.

Overall, the results from our simulation model were consistent with the original RxPONDER trial’s findings – premenopausal women benefited from chemotherapy, while postmenopausal women did not, for reasons still uncertain [6]. Further, our results are consistent with the recently published meta-analysis of clinical trials, reporting an increase in 10-year distant recurrence rates up to 20% in women with node positive breast cancer [36]. A recent study published by Stabellini et al. [10] found that chemotherapy was associated with a statistically significant improvement in overall survival regardless of age in women diagnosed with node positive (1–3), ER+, HER2 negative breast cancer, and recurrence scores of 20–25 (age < = 50: hazard ratio [HR] = 0.334, *P* = 0.002; age > 50: HR = 0.521, *P* = 0.019). Although follow-up was limited, in the RxPONDER trial for recurrences scores ≤ 25, chemotherapy showed a 1.3% absolute benefit in overall survival at 5-years in premenopausal [98.6% vs. 97.3% (HR = 0.47; *P* = 0.032)], while in postmenopausal women, these overall survival rates were 96.2% and 96.1%, respectively (HR = 0.96; *P* = 0.79) [37]. Based on these preliminary findings from the RxPONDER trial, it is unclear if overall survival benefits for chemotherapy will not vary by age in women with recurrence scores of 20–25.

In this study, we updated and validated a previously developed model [16] to evaluate the population-level, long-term effects of chemotherapy in women diagnosed with node positive, hormone receptor positive, HER2-negative breast cancer, and recurrence scores 0–25 by race and ethnicity. Simulation modeling provides a powerful computational tool to synthesize data and extrapolate clinical trial findings beyond trial follow-up for women seen in real-world practice settings. Currently, there are several methods to extrapolate trial data beyond follow-up. For example, model training and validation are commonly used in predictive modeling of cancer outcomes [38]. However, predictive modeling approaches often require adequate sample size to quantify subgroup effects and the availability of all the relevant variables in a single data source [38, 39]. In contrast, simulation modeling allowed us to combine information from several data sources to simulate larger samples and quantify the variation of chemotherapy effects in clinically relevant patient subgroups. Moreover, the use of SEER data to generate the joint distributions of patient demographic and clinical characteristics for women seen in real-world settings allowed us to generate results for racial and ethnic subgroups who were not as well represented in RxPONDER trial data. There was a persistent chemotherapy benefit in premenopausal non-Hispanic Black, Hispanic, and non-Hispanic White women, while no benefit was seen among postmenopausal women. However, despite similar treatment adherence rates modeled for the simulated women, the estimated 10-year distant recurrence-free survival rates, life-years, and QALYs across both treatment arms among non-Hispanic Black women were lower compared to the overall population. These differences could potentially be explained by the higher proportion of high grade and larger tumors at diagnosis among non-Hispanic Black women compared to the overall population. Moreover, these results are consistent with a recent retrospective analysis of the RxPONDER trial reporting lower 5-year distant relapse–free survival rates among Black women (90.1%) compared to White women (94.7%) (unadjusted HR = 1.39; *p <* 0.005) [40]. These lower rates were reported despite higher adherence rates among Black women [11].

Racial disparities in breast cancer outcomes in the U.S. have persisted since the 1980s, and our results demonstrate the persistence of these disparities [41]. Numerous factors may contribute to these disparities, including the fact that Black women may receive sub-optimal dosing and duration of chemotherapy, which is associated with obesity and related comorbidities, such as diabetes and heart disease [42–45]. However, in our study, treatment did not vary by race/ethnicity, so the worse breast cancer outcomes in non-Hispanic Black women could potentially be due to diagnostic delays that may have resulted in higher tumor grade and larger tumors at diagnoses among these women. Recent studies have shown that neighborhood deprivation and structural racism could be associated with the epigenetic landscape and tumor aggressiveness in Black women [46–50]. However, there is limited data on the impact of specific structural factors (e.g., neighborhoods) on breast cancer outcomes in node positive breast cancer. There is a need for continued research and public health investments to address these data gaps and disparities.

Our study has several limitations. Since the input parameters were derived primarily using SEER data, our results may be generalizable only to the U.S. population. Moreover, there were missing data for ER (5%), PR (5%), and HER2 (9%) status in SEER data for women diagnosed with node positive breast cancer from 2010 to 2015. We assumed these data were missing completely at random (i.e. there are no systematic differences between participants with missing data and those with complete data) [51]. The input parameters for 21-gene recurrence scores were obtained from published SEER data on the electronically linked recurrence score results from the Genomic Health Clinical Laboratory database [33]. According to published data [33], we assumed that the distribution of patient demographic and clinical characteristics in women with 21-gene recurrence scores in SEER were similar to the distributions observed in the overall population of women diagnosed with node positive (nodes 1–3), hormone response positive, HER2 negative breast cancer. Additionally, we were able to consider only three racial and ethnic subgroups. There were limited data on survival outcomes to model the effects of treatment in Asian, American Indian, or Alaska Native subgroups. The lack of representation of these subgroups in clinical trials has hindered our understanding of the variation of effects of breast cancer treatment in racial and ethnic subgroups. The utility weights from the published literature may reflect preferences of the “average” patient surveyed, but no individual is average, so it is crucial that oncologists solicit each patient’s preferences and use them to guide the treatment plan. Finally, we were not able to model the impact of structural racism including access to timely treatment, insurance, or socioeconomic status on survival outcomes. Studies quantifying the temporal effects of structural racism on DNA methylation and expression of cancer genes in breast tumors [49] could potentially support modeling the impact of racism on breast tumor biology.

## Conclusion

Despite these limitations, simulation modeling is a useful tool to extend clinical trials. Consistent with the RxPONDER trial, this simulation modeling study estimated a chemotherapy benefit among premenopausal women across all racial and ethnic subgroups and suggested that chemotherapy can be safely omitted among postmenopausal women. These results could help clinicians and patients to make informed chemotherapy decisions. These findings also highlight the need for greater racial and ethnic representation and collection of more nuanced data on structural factors in clinical trials to determine the driving forces of breast cancer disparities.

### Electronic supplementary material

Below is the link to the electronic supplementary material.

Supplementary Material 1

## Data Availability

The data generated and/or analyzed during the current study are available from the corresponding author on reasonable request.
