# Supplementary material for: The population-level effects of omitting chemotherapy guided by a 21-gene expression assay in node-positive breast cancer: a simulation modeling study
Source: BMC Cancer. 2024 Aug 8;24:975. doi: 10.1186/s12885-024-12719-3 (PMC11308572; doi:10.1186/s12885-024-12719-3)
Supplement: Supplementary file 1 — Supplementary Material 1 [file 12885_2024_12719_MOESM1_ESM.docx]

**Supplementary Methods**

The approach used to develop model inputs for this study are summarized in the manuscript, and additional details are also available from the corresponding author (JJ) on request. There was no single data source that included all the information we needed to simulate results. However, there were several good partial data sources we could use for modeling, including the published trial data^1,2^, SEER registry data,^3^ and CDC WONDER data.^4^

**The Simulation Model**

The model uses an empirical Bayesian analytic approach. The program code developed for this study was adapted from the program written for clinical trial simulation studies conducted in collaboration with the Cancer Intervention and Surveillance Modeling Network (CISNET), NCI’s Breast Oncology and Local Disease (BOLD) task force,^5^ and the TAILORx trial.^6^ We used the published time-to-event curves generated conditional on 21-gene recurrence scores, grade, tumor size, race, ethnicity, menopausal status, and age. These curves were adjusted to event rates observed in node-positive (nodes 1-3) breast cancer as described below.

***Individual Patient and Tumor Characteristics***

SEER data were used to develop joint distributions of characteristics including age, tumor grade, tumor size, race, ethnicity, and 21-gene recurrence score for women diagnosed with hormone receptor-positive (HR+), human epidermal growth factor receptor-2 (HER2)-negative, node-positive (nodes 1-3) breast cancer. We selected SEER data since our goal was to extend trial results to estimate population-level effects. This increased the generalizability of the simulated results and minimized the likelihood of bias in patient and tumor characteristics with respect to disease severity. Supplementary Table 1 summarizes the statistical models and distributions fitted into SEER data to derive the input parameters for the simulation of patient clinical and tumor characteristics. Since menopausal status was not available in SEER, we derived the joint distribution of menopausal status and age using published TAILORx data.^7^

**Supplemental Table 1.** Derivation of input parameters for age and tumor characteristics

| **Characteristics** | **Conditional on** | **Model** | **Distribution** |
| --- | --- | --- | --- |
| Age (continuous) | Race and Ethnicity | - | Cumulative distribution function |
| Tumor Grade (low, intermediate, high) | Age, Race, and Ethnicity | Multinomial logistic regression | Multinomial |
| Tumor size (≤2 cm; >2 cm) | Grade, Age, Race, and Ethnicity | Logistic regression | Binomial |
| Hormonal Sensitivity (ER or PR positive; ER and PR positive) | Grade, Race, and Ethnicity | Logistic regression | Binomial |
| 21-gene recurrence score (continuous) | Age, tumor size, grade, hormone sensitivity (and interactions), Race, and Ethnicity | Generalized linear model | Gamma/ Multinomial |
| Menopausal status | Age, Race, and Ethnicity | Logistic regression | Binomial |

***Events and Time-to-Events (Distant Recurrence and Breast Cancer Deaths)***

In a previously published study, we derived time-to-event curves using competing risk time-to-event semi-parametric models fitted to pooled trial data.^6^ The published curves were generated for time to distant recurrence conditional on age, grade, tumor size, 21-gene recurrence score, race, ethnicity, and treatment (chemo-endocrine vs. endocrine therapy) for premenopausal and postmenopausal women separately. Two-way interaction terms between race, ethnicity, 21-gene recurrence score, and age were introduced to the competing risk models to reflect the changes in the benefit of chemotherapy by these factors.^8^

The proportional-hazards cumulative incidence functions for distant recurrence were generated for reference levels of age, 21-gene recurrence scores, grade, tumor size, race, ethnicity, and interaction terms, along with estimated sub-hazard ratios (SHRs) for each attribute and covariances for those SHRs. Some of these distributions were "shrunk" by combining them with an abstract lognormal hyper-prior distribution that provided most of its support to hazard ratios between 0.25 and 4.0 consistent with published clinical trial data in early-stage breast cancer.^9^ The curves for endocrine therapy were adjusted according to the event rates observed for women diagnosed with node-positive (nodes 1-3) and hormone receptor positive breast cancer by 21-gene recurrence scores in the TransATAC trial.^10^

Simulations were conducted separately by menopausal status. Each simulation was assigned its own set of SHRs randomly drawn from the multivariate normal distribution of SHRs with those means and covariances. The replicate-specific SHRs were applied to the reference level cumulative incidence functions according to each virtual patient’s clinical and tumor characteristics and 21-gene recurrence score, race, ethnicity, and menopausal status to create an incidence function for distant recurrence for each virtual patient. The resulting incidence functions were randomly sampled to simulate times to distant recurrence. The simulated times to the distant recurrence events were a good match to the observed times in the published RxPONDER trial. A comparison of the overall simulated 5-year distant recurrence rates to the observed rates in the RxPONDER trial^11^ are provided in Table 5.

Breast cancer-specific mortality was modeled separately following the same steps described above. Breast cancer death rates beyond 10-years were extrapolated using a parametric (exponential) survival model considering women’s age, menopausal status, race, ethnicity, and treatment. Consistent with previous studies,^5,6^ we assumed that women who had a distant recurrence prior to death died due to breast cancer.

***Age and comorbidity-specific other cause mortality***

The prior distributions for race, ethnicity, and age-specific other cause survival were based on published CDC WONDER data.^4^ We added time to breast cancer-specific mortality and other cause mortality to provide simulated values for all-cause mortality. Life-years were calculated from the date of diagnosis to all-cause death for each subgroup by treatment arm.

***Simulation***

The simulation is a Monte Carlo microsimulation implemented in Stata version 18.0 (StataCorp. 2023. Stata Statistical Software: Release 18. College Station, TX: StataCorp LP.).^12^ All random sampling utilized the Mersenne Twister pseudo-random number generating algorithm in Stata version 18.

**Supplemental Table 2.** Patient and tumor characteristics of the simulated women diagnosed with node-positive (1-3 nodes), hormone receptor positive, HER2 negative breast cancer with 21-gene recurrence scores of ≤25 stratified by race, ethnicity, menopausal status, and receipt of endocrine vs. chemoendocrine therapy

| **Characteristics** | **Black (non-Hispanic)** | | **Hispanic** | | **White (non-Hispanic)** | |
| --- | --- | --- | --- | --- | --- | --- |
|  | **Endocrine therapy**  (Col %) | **Chemo- endocrine therapy**  (Col %) | **Endocrine therapy**  (Col %) | **Chemo- endocrine therapy**  (Col %) | **Endocrine therapy**  (Col %) | **Chemo- endocrine therapy**  (Col %) |
| **Overall** | | | | | | |
| **Age at Diagnosis** |  |  |  |  |  |  |
| Median, yr. | 56.3 | 56.3 | 53.6 | 53.6 | 58.0 | 58.0 |
| **Menopausal status** |  |  |  |  |  |  |
| Premenopausal | 30.9 | 30.8 | 40.7 | 40.7 | 27.0 | 27.1 |
| Postmenopausal | 69.1 | 69.1 | 59.3 | 59.3 | 73.0 | 72.9 |
| **Recurrence score** |  |  |  |  |  |  |
| Median | 17.0 | 17.0 | 16.0 | 16.0 | 17.0 | 17.0 |
| **Tumor Grade** |  |  |  |  |  |  |
| Low | 12.6 | 12.5 | 20.8 | 20.9 | 29.2 | 29.4 |
| Moderate | 61.1 | 61.3 | 57.4 | 57.4 | 57.5 | 57.4 |
| High | 26.4 | 26.2 | 21.8 | 21.7 | 13.3 | 13.2 |
| **Hormone Sensitivity** |  |  |  |  |  |  |
| ER and PR positive | 78.2 | 78.3 | 84.1 | 84.2 | 86.3 | 86.3 |
| ER or PR positive | 21.8 | 21.7 | 15.9 | 15.8 | 13.7 | 13.7 |
| **Tumor size** |  |  |  |  |  |  |
| 2 cm or less | 50.1 | 50.2 | 38.5 | 38.5 | 55.2 | 55.2 |
| Greater than 2 cm | 49.9 | 49.8 | 61.5 | 61.5 | 44.8 | 44.8 |
| **Premenopausal** | | | | | | |
| **Age at Diagnosis** |  |  |  |  |  |  |
| Median, yr. | 44.4 | 44.4 | 43.4 | 43.4 | 45.5 | 45.6 |
| **Recurrence score** |  |  |  |  |  |  |
| Median | 18.0 | 18.0 | 17.0 | 17.0 | 18.0 | 18.0 |
| **Tumor Grade** |  |  |  |  |  |  |
| Low | 10.6 | 10.6 | 15.6 | 15.6 | 26.7 | 26.8 |
| Moderate | 60.8 | 60.6 | 57.8 | 57.9 | 59.0 | 59.0 |
| High | 28.6 | 28.7 | 26.6 | 26.5 | 14.3 | 14.2 |
| **Hormone Sensitivity** |  |  |  |  |  |  |
| ER and PR positive | 75.7 | 75.6 | 83.3 | 83.3 | 84.6 | 84.5 |
| ER or PR positive | 24.3 | 24.4 | 16.7 | 16.7 | 15.4 | 15.5 |
| **Tumor size** |  |  |  |  |  |  |
| 2 cm or less | 48.8 | 48.4 | 33.6 | 33.5 | 55.4 | 55.6 |
| Greater than 2 cm | 51.2 | 51.6 | 66.4 | 66.5 | 44.6 | 44.4 |
| **Postmenopausal** | | | | | | |
| **Age at Diagnosis** |  |  |  |  |  |  |
| Median, yr. | 61.6 | 61.6 | 60.5 | 60.5 | 62.7 | 62.7 |
| **Recurrence score** |  |  |  |  |  |  |
| Median | 17.0 | 17.0 | 16.0 | 16.0 | 17.0 | 17.0 |
| **Tumor Grade** |  |  |  |  |  |  |
| Low | 13.5 | 13.3 | 24.5 | 24.4 | 30.3 | 30.2 |
| Moderate | 61.3 | 61.5 | 57.0 | 57.2 | 56.9 | 56.9 |
| High | 25.2 | 25.2 | 18.5 | 18.5 | 12.8 | 12.9 |
| **Hormone Sensitivity** |  |  |  |  |  |  |
| ER and PR positive | 79.4 | 79.4 | 84.8 | 84.8 | 87.0 | 86.9 |
| ER or PR positive | 20.6 | 20.6 | 15.2 | 15.2 | 13.0 | 13.1 |
| **Tumor size** |  |  |  |  |  |  |
| 2 cm or less | 50.7 | 50.9 | 41.8 | 41.9 | 55.1 | 55.1 |
| Greater than 2 cm | 49.3 | 49.1 | 58.2 | 58.1 | 44.9 | 44.9 |

ER: estrogen receptor; PR: progesterone receptor; HER2: Human epidermal growth factor receptor 2

**Supplemental Table 3.** Simulation model results for breast cancer death-free survival rates at 5- and 10-years for endocrine vs. chemoendocrine therapy for women diagnosed with node positive (1-3 nodes), hormone receptor positive, HER2 negative breast cancer with 21-gene-recurrence scores of ≤25, overall and stratified by menopausal status.

|  | **5-year Breast Cancer Death-Free Survival Rates (%)** | | | **10-year Breast Cancer Death-Free Survival Rates (%)** | | |
| --- | --- | --- | --- | --- | --- | --- |
|  | **Endocrine therapy** | **Chemo-endocrine therapy** | **Absolute Difference^1^** | **Endocrine therapy** | **Chemo-endocrine therapy** | **Absolute Difference^1^** |
| **Overall** | 98.1 | 97.9 | -0.2 | 91.5 | 90.7 | -0.9 |
| **Premenopausal** | 98.0 | 98.6 | 0.5 | 89.6 | 91.8 | 2.2 |
| **Postmenopausal** | 98.2 | 97.6 | -0.6 | 92.5 | 90.1 | -2.5 |

^1^Chemo-endocrine therapy (minus) Endocrine therapy Rates

**Supplemental Table 4.** Sensitivity Analysis: Simulation model results for distant recurrence-free survival at 5- and 10-years for endocrine vs. chemoendocrine therapy for women diagnosed with node positive (1-3 nodes), hormone receptor positive, HER2 negative breast cancer with 21-gene-recurrence scores of ≤25, overall and stratified by age

|  | **5-year Distant Recurrence-Free Survival Rates (%)** | | | **10-year Distant Recurrence-Free Survival Rates (%)** | | |
| --- | --- | --- | --- | --- | --- | --- |
|  | **Endocrine therapy** | **Chemo-endocrine therapy** | **Absolute Difference^1^** | **Endocrine therapy** | **Chemo-endocrine therapy** | **Absolute Difference^1^** |
| **Overall** | 93.3 | 94.5 | 1.2 | 80.5 | 83.3 | 2.9 |
| **<=50 Years** | 92.6 | 95.5 | 3.0 | 79.3 | 86.7 | 7.4 |
| **>50 Years** | 94.4 | 93.0 | - | 82.3 | 78.3 | - |

^1^Chemo-endocrine therapy (minus) Endocrine therapy Rates

**Supplemental Table 5.** Sensitivity Analysis: Simulation model results for life-years and 3% discounted quality-adjusted life-years (QALYs) for endocrine vs. chemoendocrine therapy for women diagnosed with node positive (1-3 nodes), hormone receptor positive, HER2 negative breast cancer with 21-gene-recurrence scores of ≤25, overall and stratified by age

|  | **Life Years** | | | **Undiscounted QALYs** | | | **3% Discounted QALYs** | | |
| --- | --- | --- | --- | --- | --- | --- | --- | --- | --- |
|  | **Endocrine therapy** | **Chemo-endocrine therapy** | **Life Years Gained^1^** | **Endocrine therapy** | **Chemo-endocrine therapy** | **QALYs Gained^1^** | **Endocrine therapy** | **Chemo-endocrine therapy** | **QALYs Gained^1^** |
| **Overall** | 23.7 | 24.5 | 0.8 | 13.1 | 13.0 | -0.1 | 10.6 | 10.7 | 0.1 |
| **<=50 Years** | 29.2 | 31.4 | 2.2 | 17.6 | 18.4 | 0.8 | 13.6 | 14.1 | 0.5 |
| **>50 Years** | 17.7 | 17.0 | -0.7 | 8.3 | 7.1 | -1.1 | 7.3 | 7.1 | -0.2 |

^1^Chemo-endocrine therapy (minus) Endocrine therapy Life-year or QALYs

**Supplemental Table 6.** Sensitivity Analysis: Simulation model results for varying levels of chemotherapy toxicity for 3% discounted quality-adjusted life-years (QALYs) for endocrine vs. chemoendocrine therapy for women diagnosed with node positive (1-3 nodes), hormone receptor positive, HER2 negative breast cancer with 21-gene-recurrence scores of ≤25, overall and stratified by menopausal status

|  | **Low Toxicity** | | | **High Toxicity** | | |
| --- | --- | --- | --- | --- | --- | --- |
|  | **Endocrine therapy** | **Chemo-endocrine therapy** | **QALYs Gained^1^** | **Endocrine therapy** | **Chemo-endocrine therapy** | **QALYs Gained^1^** |
| **Overall** | 11.1 | 11.2 | 0.1 | 11.1 | 11.2 | 0.1 |
| **Premenopausal** | 12.8 | 13.6 | 0.8 | 12.8 | 13.6 | 0.8 |
| **Postmenopausal** | 9.2 | 8.6 | -0.6 | 9.2 | 8.6 | -0.6 |

^1^Chemo-endocrine therapy (minus) Endocrine therapy Life-year or QALYs

**Supplemental Table 7.** Sensitivity Analysis: Simulation model results for 1 and 5% discounted quality-adjusted life-years (QALYs) for endocrine vs. chemoendocrine therapy for women diagnosed with node positive (1-3 nodes), hormone receptor positive, HER2 negative breast cancer with 21-gene-recurrence scores of ≤25 stratified by menopausal status.

|  | **1% Discounted QALYs** | | | **5% Discounted QALYs** | | |
| --- | --- | --- | --- | --- | --- | --- |
|  | **Endocrine therapy** | **Chemo-endocrine therapy** | **QALYs Gained^1^** | **Endocrine therapy** | **Chemo-endocrine therapy** | **QALYs Gained^1^** |
| **Overall** | 12.7 | 13.0 | 0.3 | 9.7 | 9.8 | 0.1 |
| **Premenopausal** | 15.0 | 16.2 | 1.2 | 11.0 | 11.6 | 0.6 |
| **Postmenopausal** | 10.2 | 9.5 | -0.7 | 8.3 | 7.8 | -0.5 |

^1^Chemo-endocrine therapy (minus) Endocrine therapy Life-year or QALYs

**Model Validation**

We used the age distribution of a trial population for model validation against the RxPONDER trial.^13^ The tumor characteristics were simulated based on the joint distributions observed in SEER. The distributions of simulated patient characteristics and published RxPONDER data are provided in Supplementary Tables 8 and 9.

**Supplemental Table 8.** Comparison of patient and tumor characteristics of the simulated and actual RxPONDER trials stratified by treatment arm.

| Characteristics | **RxPONDER** | | | |
| --- | --- | --- | --- | --- |
|  | **Actual** | | **Simulation** | |
|  | **Endocrine therapy**  **(col %)** | **Chemoendocrine therapy (col %)** | **Endocrine therapy**  **(col %)** | **Chemoendocrine therapy (col%)** |
| **Age at Diagnosis** | | | | |
| Median, yr | 57.2 | 57.9 | 56.0 | 56.0 |
| **Recurrence Score** | | | | |
| 0-13 | 42.7 | 42.9 | 25.6 | 25.8 |
| 14-25 | 57.3 | 57.1 | 74.4 | 74.2 |
| **Tumor Grade** | | | | |
| Low | 24.3 | 24.4 | 24.7 | 25.8 |
| Intermediate  /High | 74.4 | 74.4 | 75.3 | 74.2 |
| **Tumor Size** | | | | |
| 2 cm or less | 58.6 | 57.9 | 55.8 | 55.3 |
| Greater than 2 cm | 41.4 | 42.2 | 44.2 | 44.7 |

**Supplemental Table 9.** Comparison of patient and tumor characteristics of the simulated and actual RxPONDER trials stratified by menopausal status

| Characteristics | **RxPONDER** | | | |
| --- | --- | --- | --- | --- |
|  | **Actual** | | **Simulation** | |
|  | **Premenopausal**  **(col %)** | **Postmenopausal**  **(col %)** | **Premenopausal**  **(col %)** | **Postmenopausal (col%)** |
| **Recurrence Score** | | | | |
| 0-13 | 38.7 | 44.8 | 30.8 | 22.9 |
| 14-25 | 61.3 | 55.2 | 69.2 | 77.1 |
| **Tumor Grade** | | | | |
| Low | 21.7 | 25.6 | 23.2 | 27.0 |
| Intermediate/High | 77.2 | 73.0 | 76.8 | 73.0 |
| **Tumor Size** | | | | |
| 2 cm or less | 56.3 | 59.2 | 60.3 | 57.2 |
| Greater than 2 cm | 43.7 | 40.8 | 39.7 | 42.9 |

**References**

1. Paik S, Shak S, Tang G, et al. A multigene assay to predict recurrence of tamoxifen-treated, node-negative breast cancer. *N Engl J Med*. Dec 30 2004;351(27):2817-26. doi:10.1056/NEJMoa041588

2. Sparano JA, Gray RJ, Makower DF, et al. Adjuvant Chemotherapy Guided by a 21-Gene Expression Assay in Breast Cancer. *New England Journal of Medicine*. 2018;379(2):111-121. doi:10.1056/NEJMoa1804710

3. Howlader N, Altekruse SF, Li CI, et al. US incidence of breast cancer subtypes defined by joint hormone receptor and HER2 status. *Journal of the National Cancer Institute*. Apr 28 2014;106(5)doi:10.1093/jnci/dju055

4. CDC WONDER. Centers for Disease Control and Prevention. Accessed September 20, 2023. <https://wonder.cdc.gov/>

5. Jayasekera J, Li Y, Schechter CB, et al. Simulation modeling of cancer clinical trials: Application to omitting radiotherapy in low-risk breast cancer. *J Natl Cancer Inst*. Dec 1 2018;110(12):1360-1369. doi:10.1093/jnci/djy059

6. Jayasekera J, Sparano JA, Gray R, et al. Simulation Modeling to Extend Clinical Trials of Adjuvant Chemotherapy Guided by a 21-Gene Expression Assay in Early Breast Cancer (Provisional Acceptance). *Journal of the National Cancer Institute Cancer Spectrum*. 2019;

7. Sparano JA, Gray RJ, Makower DF, et al. Adjuvant Chemotherapy Guided by a 21-Gene Expression Assay in Breast Cancer. *N Engl J Med*. Jul 12 2018;379(2):111-121. doi:10.1056/NEJMoa1804710

8. Sparano JA, Crager MR, Tang G, Gray RJ, Stemmer SM, Shak S. Development and Validation of a Tool Integrating the 21-Gene Recurrence Score and Clinical-Pathological Features to Individualize Prognosis and Prediction of Chemotherapy Benefit in Early Breast Cancer. *Journal of clinical oncology : official journal of the American Society of Clinical Oncology*. Dec 11 2020:Jco2003007. doi:10.1200/jco.20.03007

9. Early Breast Cancer Trialists' Collaborative G, Peto R, Davies C, et al. Comparisons between different polychemotherapy regimens for early breast cancer: meta-analyses of long-term outcome among 100,000 women in 123 randomised trials. *Lancet*. Feb 4 2012;379(9814):432-44. doi:10.1016/S0140-6736(11)61625-5

10. Dowsett M, Cuzick J, Wale C, et al. Prediction of risk of distant recurrence using the 21-gene recurrence score in node-negative and node-positive postmenopausal patients with breast cancer treated with anastrozole or tamoxifen: a TransATAC study. *J Clin Oncol*. Apr 10 2010;28(11):1829-34. doi:10.1200/JCO.2009.24.4798

11. Sparano JA, Gray RJ, Ravdin PM, et al. Clinical and Genomic Risk to Guide the Use of Adjuvant Therapy for Breast Cancer. *N Engl J Med*. Jun 20 2019;380(25):2395-2405. doi:10.1056/NEJMoa1904819

12. StataCorp. Stata statistical software: Release 18. College Station, TX: StataCorp LLC; 2023.

13. Kalinsky K, Barlow WE, Gralow JR, et al. 21-gene assay to inform chemotherapy nenefit in node-positive breast cancer. *N Engl J Med*. Dec 16 2021;385(25):2336-2347. doi:10.1056/NEJMoa2108873
